# Did the H1N1 Vaccine Reduce the Risk of Admission with Influenza and Pneumonia during the Pandemic?

**DOI:** 10.1371/journal.pone.0142754

**Published:** 2015-11-23

**Authors:** Salaheddin M. Mahmud, Songul Bozat-Emre, Gregory Hammond, Lawrence Elliott, Paul Van Caeseele

**Affiliations:** 1 Community Health Sciences, University of Manitoba, Winnipeg, Manitoba, Canada; 2 Medical Microbiology, University of Manitoba, Winnipeg, Manitoba, Canada; 3 Medicine, University of Manitoba, Winnipeg, Manitoba, Canada; 4 Winnipeg Regional Health Authority, Winnipeg, Manitoba, Canada; 5 Cadham Provincial Laboratory, Winnipeg, Manitoba, Canada; 6 Manitoba Health, Winnipeg, Manitoba, Canada; The University of Adelaide, AUSTRALIA

## Abstract

**Background:**

The extent to which A(H1N1)pdm09 influenza vaccines prevented hospital admissions with pneumonia and influenza (P&I) during the 2009 pandemic remains poorly understood. We evaluated the effectiveness of the A(H1N1)pdm09 and seasonal influenza vaccines (TIV) used during the 2009 mass vaccination campaign in Manitoba (Canada) in preventing P&I hospitalization.

**Methods:**

A population-based record-linkage nested case-control study. Cases (N = 1,812) were persons hospitalized with influenza (ICD-10:J09-J11) or pneumonia (ICD-10:J12-J18) during the study period. Age-, gender- and area of residence-matched controls (N = 7,915) were randomly sampled from Manitoba’s Population Registry. Information on receipt of A(H1N1)pdm09 vaccine and TIV was obtained from the Manitoba Immunization Monitoring System, a province-wide vaccine registry.

**Results:**

Overall, the adjuvanted A(H1N1)pdm09 vaccine was 27% (95%CI 13–39%) effective against P&I hospitalization ≥ 14 days following administration. Effectiveness seemed lower among older (≥ 65 years) adults (10%; −16–30%), particularly when compared to under-5 children (58%; 30–75%). The number-needed-to-vaccinate to prevent 1 P&I admission was lowest among <4 year-olds (928) and ≥65 years (1,721). VE against hospitalization with laboratory-confirmed A(H1N1)pdm09 was 70% (39–85%) overall and (91%; 62–98%) ≥ 14 days following vaccination.

**Discussion:**

Our data suggest that the adjuvanted A(H1N1)pdm09 vaccine was effective in preventing about 55–60% of P&I hospitalizations among children and younger adults who were at much higher risk of infection. Unfortunately, the vaccine was less effective among 65 or older adults. Despite that the vaccine still had a significant population-based impact especially among the very young (<5) and the older (≥ 65 years).

**Key Points**

In the recent pandemic, the **A(H1N1)pdm09 vaccine** was effective in preventing 55–60% of **influenza and pneumonia** hospitalizations among children and younger adults. Although less effective among ≥65 adults(~10%), the vaccine benefited this group the most as measured by the number-needed-to-vaccinate.

## Introduction

Worldwide, more than 26 different monovalent A(H1N1)pdm09 vaccines were used during the 2009 influenza pandemic to control the spread of infection and reduce disease burden[1]. In Manitoba (Canada), we found that both adjuvanted and non-adjuvanted vaccines were effective in preventing laboratory-confirmed A(H1N1)pdm09 infections[2], and similar findings were reported for other jurisdictions.[3] However, the extent to which vaccination prevented more clinically meaningful outcomes, such as acute hospital admission due to influenza and pneumonia (P&I), is not known.

Rates of laboratory-confirmed influenza can vary significantly between jurisdictions and with epidemic phase in the same jurisdiction as a function of clinical guidelines, epidemic phase and laboratory workload. By comparison, decisions for hospitalization are less discretionary, and as a result hospitalization rates are a robust indicator of epidemic severity.[4] Arguably, avoiding hospitalization is a more relevant outcome for influenza control efforts than the mere reduction in numbers of laboratory-confirmed infections.

We evaluated the effectiveness of the A(H1N1)pdm09 and seasonal influenza vaccines (TIV) used during the 2009 mass vaccination campaign in Manitoba (Canada) in preventing P&I hospitalization using a case-control design and data from Manitoba’s provincial administrative and laboratory databases. We also assessed vaccine effectiveness (VE) for different age groups and among high-risk populations, e.g., immunocompromised persons.

## Methods

### Design

We conducted a nested case-control study using de-identified records obtained by linking the electronic database of the Manitoba Immunization Monitoring System (MIMS) with the Hospital Separation Abstract database and other Manitoba Health (MH) administrative databases, housed at the Manitoba Centre for Health Policy. MH provides publicly-funded universal healthcare insurance to virtually all of Manitoba’s 1.2 million residents regardless of age or income [5]. Insured services include hospital, physician and preventive services (e.g., immunizations). For administrative purposes, MH maintains several centralized electronic databases that can be linked using a unique health services number. The study was approved by the Research Ethics Board of the University of Manitoba and the governmental Health Information Privacy Committee.

### Definition of cases and controls

Anyone 6 months or older who was registered with MH during the study period was eligible for inclusion in the study. The study spanned the period from November 2, 2009 (1 week after the start of mass immunization in Manitoba) to February 7, 2010, two weeks following the last reported A(H1N1)pdm09 case in the province.

Any eligible person who was admitted for ≥24 hours to a hospital in Manitoba with a diagnosis of influenza (ICD-10: J09-J11) or pneumonia (ICD-10: J12-J18) during the study period was included in the cases group. Cases were identified using the Hospital Separation Abstract database, which, since 1971, has recorded all services provided by hospitals in the province, including admissions and day surgeries [6]. The data collected include clinical information such as admission and discharge dates and up to 25 diagnoses and 20 services or procedures, coded using the International Classification of Diseases (ICD-10-CA[7]) and the Canadian Classification of Health Interventions (CCI)[8].

Using risk-set sampling, we matched each case to five controls (persons who have not been admitted to hospital by the index date) who were of the same age, gender and area of residence. The date of admission was considered as the “index date” for cases and for their matched controls. Controls were randomly selected from MH’s Population Registry, a continuously updated database that tracks dates and reasons for the initiation and termination of coverage (e.g., death/migration) for all insured persons.

### Determination of vaccination status

For all cases and controls, information on the receipt of the pandemic, seasonal influenza and pneumococcal vaccines during and before the 2009/10 season was obtained from MIMS, a population-based province-wide registry of virtually all vaccines administered to Manitoba residents since 1988 [9]. Information, including vaccine type and date of vaccination, is captured either through direct data entry for vaccines administered by public health staff (who administered most influenza vaccines during the pandemic) or using physician claims data for vaccines administered by physicians [10].

In Manitoba, most pandemic vaccines were administered during a mass immunization campaign that began in October 26, 2009 (Week 43), just one week before the peak of the second pandemic wave [2]. Initially, the Canadian-manufactured Arepanrix^®^ (GlaxoSmithKline), an AS03-adjuvanted split virion monovalent vaccine, was used to vaccinate adults and children over 6 months of age. Later on, two nonadjuvanted vaccines, from GlaxoSmithKline and CSL Limited, were offered to pregnant women and children 10 years or older [11]. All vaccines contained the A(H1N1)pdm09 hemagglutinin antigen derived from the influenza A/California/7/2009 strain recommended by the WHO. A single vaccine dose (15μg/0.5 ml) was recommended for those aged >9 years and 2 half doses given 21 days apart were recommended for children 6 months-9 years old.

All vaccines were offered free of charge, but due to limited supply at campaign start priority was given to certain groups including health care workers, Aboriginal persons, residents of remote communities, pregnant women, 6–60 months-old children, persons under 65 years with chronic medical conditions and all immunocompromised persons. On November 18, 2009, vaccines became available to the whole population [2].

Manitoba’s routine immunization schedule includes seasonal trivalent inactivated influenza vaccines (TIV)—during the study period these were Fluviral^®^ (GlaxoSmithKline) and Vaxigrip^®^ (Sanofi Pasteur)—and several polysaccharide and conjugate pneumococcal (PVs).

### Potential confounders

Individuals were assigned to a neighbourhood of residence (neighbourhood clusters within the capital city of Winnipeg and regional health authorities in the rest of the province) based on their postal code as recorded in MH’s Population Registry. Household income quintiles, measured at the level of Census Dissemination areas, were determined using 2006 Canadian census data. Information on pregnancy, comorbidities, propensity to seek health care (measured as the number of hospital and family physician visits in the previous 5 years) was obtained from the Hospital Separation and Physician Claims databases. Previously validated algorithms were used to identify various chronic diseases and other indications for vaccination [12–15]. In addition, Charlson comorbidity scores were calculated using an algorithm validated for administrative databases[16]. Information on the use of antivirals and other medications was obtained from the Drug Program Information Network database, the comprehensive database of all out-of-hospital prescriptions dispensed in Manitoba since 1995 [17].

Influenza testing results were obtained from the database of Cadham Provincial laboratory (CPL), the province’s only public health laboratory. During the study period, influenza testing in Manitoba was completed at CPL using a real-time multiplex reverse-transcription polymerase chain reaction (RT-PCR) assay developed by the National Microbiology Laboratory [18].

### Statistical analysis

In the primary analysis, we used conditional logistic regression models to estimate the odds ratio (OR) for the association between the receipt of the adjuvanted A(H1N1)pdm09 vaccine and P&J hospitalization while adjusting for matching and confounding variables. Models were adjusted for income, comorbidity, receipt of seasonal and pneumococcal vaccines, use of neuraminidase inhibitors, frequency of contact with healthcare providers and belonging to a vaccine priority group. VE was estimated as (1-OR) x 100. Similar but separate models were used to estimate the VE of nonadjuvanted pandemic, seasonal and pneumococcal vaccines.

In primed healthy adults, the peak serum antibody levels are typically observed >2 weeks after vaccination [19]. To account for differences in effectiveness by time since vaccination, we classified vaccinated individuals into three groups depending on whether vaccination occurred 1–6, 7–13, or ≥ 14 days before the index date, and contrasted the odds of A(H1N1)pdm09 infection in each group with the odds of infection among the unvaccinated.

In addition, we repeated the adjuvanted A(H1N1)pdm09 vaccine analyses after stratifying by age group, place of residence, epidemic phase (admission before and after the peak), presence of high-risk conditions and belonging to a vaccine priority group. We also assessed for possible effect modification by use of seasonal and pneumococcal vaccines. The statistical significance of adding the interaction terms was assessed using the likelihood ratio test [20].

## Results

A total of 1,812 persons met the case definition and were matched to 9,060 controls. About 54% of cases were 65 years or older at diagnosis, and 39% resided in the poorest parts of the province (Table 1). As expected, cases tended to be generally sicker than controls with higher Charlson comorbidity scores (average score of 3 compared to 0 for controls), higher overall chance of having ≥1 diagnosed chronic medical conditions (73% compared to 39% among controls) and more frequent hospitalizations and physician consultations. As a result, cases were also more likely to belong to A(H1N1)pdm09 or TIV priority groups (Table 1).

**Table 1:** Demographic and clinical characteristics of cases and controls.

Variables	Cases		Controls		
	N	%	N	%	Totals
*Total*	*1*,*812*	*16*.*7*	*9*,*060*	*83*.*3*	*10*,*872*
Age group (years)					
0.5–4	161	8.9	770	8.5	931
5–24	130	7.2	668	7.3	798
25–44	171	9.4	859	9.5	1,030
45–64	369	20.4	1,892	20.9	2,261
≥65	981	54.1	4,871	53.8	5,852
Female	899	49.6	4,495	49.6	5,394
Resides in an urban area	932	51.4	4,660	51.4	5,592
Resides in northern Manitoba	169	9.3	845	9.3	1,014
Quintiles of household income					
Q1 (lowest)	707	39.0	3,034	33.5	3,741
Q2	407	22.5	1,967	21.7	2,374
Q3	306	16.9	1,700	18.8	2,006
Q4	231	12.7	1,242	13.7	1,473
Q5 (highest)	161	8.9	1,117	12.3	1,278
In a high priority group for the A(H1N1)pdm09 vaccine	716	39.5	2,125	23.5	2,841
Recommended recipient of the 2009/10 seasonal influenza vaccine	1,554	85.8	6,296	69.5	7,850
Pregnant (% of all 15–49 old women)	31	18.7	18	2.2	49
Has a chronic disease*	1,290	71.2	3,507	38.7	4,797
Mean Charlson index (SD)	3	2.7	0	1.2	1
Asthma	232	12.8	320	3.5	552
Chronic obstructive pulmonary disease	590	32.6	729	8.0	1,331
Ischemic heart diseases	532	29.4	1,303	14.4	1,835
Cancer (excluding non-melanoma skin cancer)	410	22.6	1,097	12.1	1,507
Chronic renal failure	206	11.4	174	1.9	380
Diabetes	526	29.0	1,305	14.4	1,831
Stroke	137	7.6	276	3.0	413
Immunosuppressed	581	32.1	1,309	14.4	1,890
≥1 hospital admissions in the last 5 years	1,219	67.3	3,113	34.4	4,332
≥20 physician encounters in the last 5 years	1,440	79.5	5,462	60.3	6,902
Received antibiotics	509	28.1	533	5.9	1,042
Tested for A(H1N1)pdm09	921	49.8	6	<0.1	927
Tested positive for A(H1N1)pdm09	149	8.2	0	0.0	149

SD: standard deviation.

* Defined as diagnosis with one of following diseases: diabetes, chronic obstructive pulmonary disease, asthma, ischemic heart disease, chronic renal failure, or cancer (excluding non-melanoma skin cancer).

A similar proportion of cases and controls received the adjuvanted A(H1N1)pdm09 vaccine (about 35%; Table 2), comparable to estimates of vaccine coverage for the entire population from MH (37% [11]) and the 2010 Canadian Community Health Survey (37% [95%CI: 33–41%])[21]. However, only about 28% of both groups received the vaccine ≥14 days before the index date corresponding to a VE estimate (adjusted for matching covariates) of 0.2% (95%CI: −13–12%). After adjusting for important confounders, the corresponding VE estimate was 27% (13–39%). Because of small numbers it was not possible to reliably estimate VE of the nonadjuvanted vaccines. In adjusted models, neither receiving the TIV in the 2008/09 or 2009/10 seasons nor receiving a pneumococcal vaccine at any point before the index date had a significant influence on the risk of hospitalization during the study period (Table 2).

**Table 2:** Estimates of the effectiveness (VE) of pandemic, seasonal influenza and pneumococcal vaccine against hospitalization due to influenza or pneumonia*.

			Model A**			Model B***		
Variable	Cases	Controls	VE	95% CI	P-value	VE	95% CI	P-value
*Total*	*1*,*812*	*9*,*060*						
**Received an adjuvanted A(H1N1)pdm09 vaccine**								
Any time before the index date	610 (33.7)	3,158 (34.9)	5.9	-5.6–16.2	0.303	27.1	14.4–37.9	< .001
1–6 days before the index date	50 (2.8)	314 (3.5)	24.1	-4.4–44.8	0.090	24.1	-12.3–48.7	0.167
7–13 days before the index date	54 (3.0)	325 (3.6)	20.2	-8.3–41.3	0.148	26.3	-8.9–50.1	0.126
≥14 days before the index date	506 (28.0)	2,519 (27.8)	0.2	-13.4–12.2	0.971	27.3	13.2–39.2	< .001
**Received a nonadjuvanted A(H1N1)pdm09 vaccine**								
Any time before the index date	10 (0.6)	41 (0.5)	-20.3	-141.8–40.2	0.604	-11.5	-212.8–60.2	0.836
**Received a seasonal influenza vaccine (TIV)**								
In the 2008/09 season	771 (42.5)	3,562 (39.3)	-19.9	-35.1–-6.4	0.003	-5.7	-24.6–10.4	0.514
In the 2009/10 season								
Any time before the index date	744 (41.1)	3,421 (37.8)	-20.3	-35.5–-6.8	0.002	-0.6	-19.1–15.0	0.945
1–13 days before the index date	49 (2.7)	254 (2.8)	-2.9	-42.3–25.6	0.863	7.4	-43.1–40.1	0.729
≥14 days before the index date	695 (38.4)	3,167 (35.0)	-22.2	-38.3–-8.1	0.001	-1.5	-20.9–14.7	0.866
**Received a pneumococcal vaccine**								
Any time before the index date	429 (23.7)	1,849 (20.4)	-35.3	-57.2–-16.4	< .001	8.3	-12.4–25.2	0.404

*In these analyses, individuals vaccinated before the identified time duration considered unvaccinated

**Model A: Adjusted for age, gender, place of residence;

***Model B: Adjusted for Model A variables plus income, comorbidity, A(H1N1)pdm09 priority group, receiving the 2009/10 seasonal influenza vaccine, receiving a pneumococcal vaccine, immunosuppressed, pregnancy, ≥20 physician encounters in the last 5 years, ≥1 hospital admission in the last 5 years; use of antiviral prophylaxis and diagnosis of chronic renal failure.

In subgroup analyses (Table 3), there was evidence that the adjuvanted A(H1N1)pdm09 vaccine was less effective in preventing P&I hospitalization among 65 or older adults (10% [−16–30%]) compared to younger age groups, particularly under-5 children (58% [30–75%]). The A(H1N1)pdm09 vaccine was also less effective among those with chronic diseases (14% [−11–34%]; *P*_interaction_ = 0.044) and among those who received the 2008/09 TIV (21% [1–37%]), although the latter finding was not statistically significant (*P*_interaction_ = 0.3). On the other hand, there was a statistically significant difference (*P*_interaction_ = 0.016) in A(H1N1)pdm09 VE between those who also received the 2009/10 TIV (12% [−21–36%]) and those who did not (46% [28–60%]).

**Table 3:** Estimates of the effectiveness (VE) of the adjuvanted A(H1N1)pdm09 vaccine (when received ≥14 days before the index date) against hospitalization due to influenza or pneumonia by certain demographic and clinical characteristics.

			Model A**			Model B***		
Variable	Cases	Controls	VE	95% CI	P-value	VE	95% CI	P-value
*Total*	*1*,*698*	*7*,*915*						
**Age group (years)**								
0.5–4	142	607	38.1	7.2–58.7	0.020	58.1	30.4–74.8	< .001
5–24	115	536	-1.1	-82.8–44.1	0.972	53.7	-15.9–81.5	0.100
25–44	163	757	5.3	-47.9–39.4	0.809	25.8	-46.0–62.3	0.388
45–64	349	1,657	-41.0	-87.6–-6.0	0.018	40.7	-0.4–65.0	0.052
≥65	929	4,358	0.0	-19.0–16.1	0.996	9.9	-15.6–29.7	0.414
P for interaction					0.019			0.415
**Chronic disease** *								
Yes	1,211	3,100	7.5	-10.5–22.6	0.389	14.4	-11.1–34.0	0.242
No	487	4,815	41.6	23.2–55.7	< .001	44.5	21.6–60.7	< .001
P for interaction					0.030			0.044
**Immunosuppressed**								
Yes	554	1,159	-3.8	-47.5–26.9	0.835	-6.3	-82.2–38.0	0.824
No	1,144	6,756	15.9	0.8–28.7	0.039	37.1	20.7–50.1	< .001
P for interaction					0.100			0.128
**In a high priority group for the A(H1N1)pdm09 vaccine**								
Yes	661	1,761	8.0	-16.2–27.1	0.485	35.9	11.8–53.4	0.006
No	1,037	6,154	6.6	-10.7–21.2	0.429	16.0	-6.5–33.8	0.150
P for interaction					0.632			0.811
**Ever received a pneumococcal vaccine**								
Yes	388	1,538	32.0	6.0–50.8	0.020	48.9	22.3–66.4	0.002
No	1,310	6,377	-1.5	-18.6–13.1	0.853	21.5	1.5–37.4	0.036
P for interaction					0.396			0.191
**Received the 2008/09 seasonal influenza vaccine**								
Yes	706	3,088	13.0	-8.8–30.5	0.222	22.7	-6.0–43.6	0.110
No	992	4,827	4.9	-16.5–22.4	0.625	40.6	20.7–55.4	< .001
P for interaction					0.608			0.314
**Received the 2009/10 seasonal influenza vaccine**								
Yes	681	2,961	4.4	-20.7–24.3	0.705	12.2	-21.1–36.3	0.428
No	1,017	4,954	21.9	2.5–37.4	0.029	46.2	28.1–59.7	< .001
P for interaction					0.017			0.016

*Defined as diagnosis with one of following diseases: diabetes, chronic obstructive pulmonary disease, asthma, ischemic heart disease, chronic renal failure, or cancer (excluding non-melanoma skin cancer).

**Model A: Adjusted for age, gender, place of residence;

***Model B: Adjusted for Model A variables plus income, comorbidity, A(H1N1)pdm09 priority group, receiving the 2009/10 seasonal influenza vaccine, receiving a pneumococcal vaccine, immunosuppressed, pregnancy, ≥20 physician encounters in the last 5 years, ≥1 hospital admission in the last 5 years; use of antiviral prophylaxis and diagnosis of chronic renal failure.

About 8% of the cases and none of the controls tested positive for A(H1N1)pdm09 (Table 4), which is not surprising given provincial guidelines discouraging viral testing unless the patient is very ill. Only 6 controls were tested within 2 weeks of the index date compared to 921 cases. In analyses limited to cases hospitalized with laboratory-confirmed A(H1N1)pdm09, 13% of the cases received an adjuvanted A(H1N1)pdm09 vaccine compared to 25% of the controls (Table 4), corresponding to a VE estimate (adjusted for matching covariates) of 59% (30–76%). The corresponding fully adjusted estimate was 70% (39–85%). VE among those who were vaccinated ≥ 14 days before the index date was higher (91%; 62–98%) than among those who were vaccinated <7 days (46%; −48–81%) or 7–13 days before the index date (61%; −11–86%). Despite small numbers, estimates of VE of the nonadjuvanted A(H1N1)pdm09 vaccine were comparable. On the other hand, there was no evidence that the 2008/09 or the 2009/10 TIVs reduced the risk of hospitalization with laboratory-confirmed A(H1N1)pdm09.

**Table 4:** Estimates of the effectiveness (VE) of pandemic, seasonal influenza and pneumococcal vaccine against hospitalization with laboratory-confirmed influenza*.

			Model A**			Model B***		
Variable	Cases	Controls	VE	95% CI	P-value	VE	95% CI	P-value
*Total* *	*149*	*745*						
**Received an adjuvanted A(H1N1)pdm09 vaccine**								
Any time before the index date	19 (12.8)	186 (25.0)	58.7	30.2–75.6	< .001	70.2	39.4–85.4	< .001
1–6 days before the index date	7 (4.7)	43 (5.8)	31.6	-63.5–71.3	0.393	46.3	-47.8–80.5	0.229
7–13 days before the index date	8 (5.4)	48 (6.4)	29.7	-53.7–67.8	0.377	60.7	-11.3–86.2	0.079
≥14 days before the index date	<6^†^ (<4.0)	95 (12.8)	83.4	53.5–94.1	< .001	91.1	62.3–97.9	< .001
**Received non-adjuvanted A(H1N1)pdm09 vaccine**								
Any time before the index date	<6 (<4.0)	8 (1.1)	-1.6	-382.6–78.6	0.984	97.5	47.2–99.9	0.018
**Received a seasonal influenza vaccine (TIV)**								
In the 2008/09 season	28 (18.8)	108 (14.5)	-44.4	-137.6–12.2	0.148	-41.5	-178.7–28.1	0.315
In the 2009/10 season								
Any time before the index date	24 (16.1)	89 (11.9)	-49.4	-152.7–11.7	0.134	-28.4	-182.3–41.6	0.534
1–13 days before the index date	<6 (<4.0)	25 (3.4)	-7.9	-190.6–59.9	0.880	-86.9	-672.7–54.8	0.388
≥14 days before the index date	19 (12.8)	64 (8.6)	-68.2	-204.5–7.0	0.086	-15.4	-175.0–51.5	0.746
**Received a pneumococcal vaccine**								
Any time before the index date	26 (17.4)	118 (15.8)	-42.3	-219.1–36.5	0.392	25.4	-103.0–72.6	0.567

*In these analyses, individuals vaccinated before the identified time duration considered unvaccinated

**Model A: Adjusted for age, gender, place of residence;

***Model B: Adjusted for Model A variables plus income, comorbidity, A(H1N1)pdm09 priority group, receiving the 2009/10 seasonal influenza vaccine, receiving a pneumococcal vaccine, immunosuppressed, pregnancy, ≥20 physician encounters in the last 5 years, ≥1 hospital admission in the last 5 years; use of antiviral prophylaxis and diagnosis of chronic renal failure.

^†^ Exact numbers between 1–5 are not reported as required by the data custodian to protect patient confidentiality.

## Discussion

Our data suggest that the adjuvanted A(H1N1)pdm09 vaccine prevented about 55 to 60% of P&I hospitalizations among children and younger adults and a much lower percentage (10–15%) of hospitalizations among 65 or older adults and among those with pre-existing chronic diseases (14%). The vaccine was also effective (70% on average) in preventing hospitalizations with laboratory-confirmed A(H1N1)pdm09 with higher levels of protection achieved >14 days after vaccination.

Excellent immune responses following even one dose of the monovalent split/subunit inactivated pandemic vaccines were documented in several immunogenicity trials [22, 23]. In post-marketing studies, the vaccines were also effective in preventing laboratory-confirmed A(H1N1)pdm09 infections during the pandemic. In a systematic review that was limited to 5 observational studies which met stringent quality criteria, the median VE of monovalent A(H1N1)pdm09 vaccines was about 69% [3]. Similar estimates were observed in studies not included in the review [2, 24].

To our knowledge, this is the first published study to evaluate the effectiveness of the A(H1N1)pdm09 vaccine against admission with P&I during the pandemic. On the other hand, VE against hospitalization with A(H1N1)pdm09 infection was examined in few studies. Using data from a Scottish general practice sentinel surveillance network, Simpson et al reported that the adjuvanted vaccine was 95% (76–100%) effective against laboratory-confirmed A(H1N1)pdm09 [25]. Emborg, et al. estimated, using Danish health databases, that the adjuvanted monovalent vaccine was 44% (−19%–73%) effective in preventing hospitalization with laboratory-confirmed A(H1N1)pdm09 infection among younger (<65) chronically ill people [26]. Steens, et al. found that among persons with underlying medical conditions or ≥ 60 years of age, a single dose of the MF-59 adjuvanted A(H1N1)pdm09 vaccine had VE of 19% (−28–49%) [27].

Higher estimates of VE against P&I hospitalization among children and young adults in our study may reflect the greater contribution of A(H1N1)pdm09 to P&I hospitalization during the pandemic among this age group compared to older adults who generally were less likely to become infected [28, 29]. Also, in a previous analysis from Manitoba we found that the vaccine was more effective in preventing A(H1N1)pdm09 infection in children compared to older adults.[2] This is also consistent with studies that examined VE against hospitalization with A(H1N1)pdm09 among children. In a Quebec study, VE of a single pediatric dose of the same AS03-adjuvanted vaccine that was used in Manitoba, was 85% (61–94%) among 6 month-9 year olds and slightly lower in 5–9 year-olds at 79% (−31–96%) [30]. In a smaller study conducted in New York, a single dose of the nonadjuvanted vaccine was 82% (0–100%) effective in in preventing hospitalization ≥ 14 days after vaccination in children aged 3–9 years [31]. Based on this evidence, it is reasonable to conclude that that the A(H1N1)pdm09 was effective in protecting younger children against hospitalization with P&I during the pandemic.

For older adults, our A(H1N1)pdm09 VE estimates were lower than estimates obtained from observational studies of the effectiveness of TIVs against P&I hospitalization during non-pandemic seasons. In a comprehensive Cochrane review,[32] 8 such studies had a pooled estimate of 26% (12%-38%) during seasons when the vaccine was well matched to the circulating strain. However, our estimates are more in line with the studies that attempted to control for confounding by the “healthy vaccinee effect” (seniors who get vaccinated are on average healthier than those who do not) which produced estimates between 8 and 14%[33–35].

Despite lower VE and lower incidence of A(H1N1)pdm09 among the elderly, our data suggest that only very young children (0–4 years) had a lower number needed to vaccinate (NNV = 928) to prevent one hospital admission for P&I. The NNV among 65 or older was 1,721 compared to 2,273 among 45–64 olds and 7,598 among 25–44 olds. This reflects the much lower overall rate of P&I admissions among the latter two groups (4–5/10,000) compared to 59/10,000 among the ≥65 age group and 19/10,000 among 0–4 year olds. Ignoring potential indirect benefits due to herd immunity, these figures suggest that vaccinating the <5 and ≥65 age groups was more cost-effective than vaccinating other groups.

In our study, the TIVs did not appear to protect against P&I admissions overall or due to A(H1N1)pdm09. This makes sense because in Manitoba the pandemic strain almost entirely replaced previously circulating influenza strains during the 2009/10 season and there was no evidence of significant cross-protective response in previous studies.[36] We observed that the A(H1N1)pdm09 vaccine was less effective among those who also received the 2009/10 TIV. It is unclear whether this reflects a biological effect or confounding by indication because the TIV is indicated to persons at higher risk of developing severe influenza illness and its complications [37]. The issue of whether TIV use before or during the pandemic increased the incidence or severity of A(H1N1)pdm09 remains controversial [38].

## Strengths and Limitations

Because of its population-based design and the availability of accurate automated hospital admission and vaccination records, this study is less susceptible to selection and recall biases that commonly afflict conventional case-control studies [10]. Because cases and controls were identified on the basis of comprehensive hospital records with high standards of coding practices,[13] misclassification of hospitalization status is also not a major concern in this study.

We used proxies for access to health care (e.g., frequency of physician encounters) to adjust for factors associated with the likelihood of admission and influenza testing. We also adjusted for confounding by several vaccine indications such as immune status and pre-existing health conditions using information obtained from administrative databases. The completeness and accuracy of the MH database are well established [6, 39], and these databases have been used extensively in studies of post-marketing surveillance of drugs and vaccines [2]. However, it is still possible that some variables were measured with error, which could result in residual confounding. We did not have information on functional status. However, the observed protective effects of A(H1N1)pdm09 vaccination are unlikely to be due to the healthy vaccinee effect, because vaccination was targeted at the higher-risk, and generally less healthy, groups, and we adjusted for vaccine indications in our models. Also, we did not observe any protective effects for the TIVs which are presumably subject to the same bias.

Confounding by herd immunity following the summer wave of the pandemic is also unlikely explanation for our findings, because we adjusted for area of residence in all models and because VE estimates were similar for northern and southern communities despite significant differences in A(H1N1)pdm09 seroprevalence between these communities at the end of the summer wave of the pandemic [28].

## Conclusions

Our data suggest that the adjuvanted A(H1N1)pdm09 vaccine was effective in preventing about 55–60% of P&I hospitalizations among children and younger adults who were at much higher risk of infection. Unfortunately, the vaccine was less effective among 65 or older adults. Despite that the vaccine still had a significant population-based impact especially among the very young (<5) and the older (≥ 65 years).
